# Health care professionals’ perspectives on barriers to treatment seeking for formal health services among orphan children and adolescents with HIV/AIDS and mental distress in a rural district in central, Uganda

**DOI:** 10.1186/s13034-020-00332-8

**Published:** 2020-06-03

**Authors:** James Mugisha, Eugene Kinyanda, Joseph Osafo, Winfred Nalukenge, Birthe Loa Knizek

**Affiliations:** 1 Kyambogo University, Kampala, Uganda; 2 Butabika National Referral and Teaching Mental Hospital, Kampala, P.O. Box 2958, Kampala, Uganda; 3 MRC/UVRI & LSHTM Uganda Research Unit On AIDS & Senior Wellcome Trust Fellowship, 50-59 Nakiwogo Street, Entebbe, Uganda; 4 Department of Psychiatry, Makerere University, Mulago Hill, Kampala, Uganda; 5 College of Humanities, School of Social Sciences, Department of Psychology, University of Ghana, Legon, Accra, Ghana; 6 Faculty of Medicine and Health Sciences, Norwegian University of Science and Technology, Trondheim, Norway

**Keywords:** Children, HIV/AIDS, Mental distress, Uganda

## Abstract

**Background:**

Little/no research has been conducted in Uganda in particular and sub-Saharan Africa in general on the health professional’s perspectives on barriers to treatment seeking for formal health services among orphan children and adolescents with a double burden of HIV/AIDS and mental distress.

**Aim:**

To explore health professionals’ perspectives on barriers to treatment seeking for formal health services among orphan children and adolescents with HIV/AIDS and mental distress in Masaka, Uganda.

**Method:**

Qualitative research design using key informant interviews with health service managers and staff in agencies working with children and adolescents with HIV/AIDS in Masaka district, Uganda.

**Results:**

Barriers to treatment seeking reported by health care professionals were quite enormous and are summarized under: family, individual, community and health systems level barriers. The crosscutting finding here is that the societal informal and formal systems of care had been affected by the HIV/AIDs epidemic, and, mental distress aggravates this challenge for the individuals afflicted and families affected by mental distress.

**Conclusion:**

Children and adolescents with both HIV/AIDS and mental distress are vulnerable due to constraints at family, community and health systems levels. Effective public health interventions to address the double burden of HIV/AIDS and mental distress will be vital in the study communities addressing the constraints at family, community and institutional level. Public health interventions should aim at increased access and effective utilization of services for both HIV/AIDS and mental health services. Stigma reduction strategies at individual, family and community levels are also recommended.

## Background

There are unprecedented efforts in Africa in general and Uganda in particular to expand care for children with HIV/AIDS [1]. A large number of these children are orphans. Health care professionals are a critical resource in HIV/AIDS care and in advising government and private sector agencies on how to improve treatment seeking for children and adolescents seeking HIV/AIDS services (including HIV/AIDS orphans) in the country [2].

Globally, in 2015, there were around 140 million orphans with around 52 million in Africa [3]. In 2014, Uganda as a country was estimated to have around 1.4 million orphans [4] while the Uganda Demographic and Health Survey [5] estimated that almost one [1] in seven [7] children under age eighteen [18] is orphaned. The largest proportion of these children has been orphaned by HIV/AIDS [6, 7]. It has been estimated that Uganda has 200,000 children that are HIV infected. Of these, 70,000 (35%) are on ART (antiretroviral therapy), with the remaining number expected to start ART in the near future [1]. In terms of adolescents, HIV is the second leading cause of death among adolescents in Uganda [3].

The association between mental health and poverty in low-income countries is increasing and well established [8, 9]. Poverty and deprivation are known to significantly constitute and exacerbate mental distress [8]. Poor communities are exposed to stressors that are social, economic, and psychological [8]. In the study area, due to poverty, the children are likely to face conditions such as hunger, neglect, violence, stigma and homelessness. In this study, we hold the view that a range of these conditions contributes to mental distress, which might be a prodromal period and therefore must be taken seriously [8]. We therefore take a broader view to mental health as opposed to the largely existing focus in mental health research on clinically defined mental disorders [9]. The narratives in this study should therefore be interpreted as referring to subjectively experienced distress, which might develop into more serious clinical conditions. Data on the entire burden of mental distress among children and adolescents in Uganda is not available at the moment. The few related studies that have been conducted in Uganda among HIV/AIDS study populations have had a biased view of mental distress (some assessed the prevalence of emotional and behavioral disorders while others had their focus on the prevalence of mental disorders). Musisi and Kinyanda [10] studied behavioral and emotional disorders among children and adolescents that have suffered the brunt of HIV/AIDS. They reported significant psychological distress (more than half of their respondents had psychological distress). For example, 45% orphans as compared to 36% non-orphans had psychological distress [10]. The same study reported that neither orphans nor non-orphans had major psychiatrist disorders (such as psychosis, major affective organic syndromes) but both groups exhibited significant emotional and behavioral problems. In another related study, Nalugya and colleagues [11] assessed psychiatrist problems among school-going adolescents in central Uganda. They reported that 21% of their participants had significant symptoms of depression while 8% met the criteria for major depression. Other related studies conducted in northern Uganda, an area that suffered civil strife, investigated the burden of mental disorders and also unraveled a high burden of these conditions among children and adolescents [12–15].

Given the high burden of HIV/AIDS and mental distress in Uganda among children and adolescents due some of the risk factors mentioned above, health care professionals’ beliefs and perspectives on barriers and facilitators for treatment seeking for HIV/AIDS services for orphan children are vital today. They play an important gate-keeping role in the health service sector and their information is vital in the design of HIV/AIDS and mental health services.

To the best of our knowledge, the current study is the first to be conducted in Uganda, Africa’s sub-Saharan region, on health care professionals perspectives on treatment seeking barriers for formal health services among orphans and adolescents with HIV/AIDS and mental distress.

## Methods

### Design

This was an exploratory qualitative study design. The study is deemed exploratory since there are no similar studies in Uganda that have been conducted on health care professionals perspectives on barriers to treatment seeking for HIV/AIDS.

### Study setting

This study is part of a longitudinal study among children and adolescents with HIV in both urban and rural Uganda: ‘*Mental health among HIV infected CHildren and Adolescents in KAmpala and Masaka, Uganda (CHAKA)*’. Kampala is the capital city of Uganda with a population of around 1.7 million people. It has a relatively better social service sector as compared to most rural areas in Uganda. Masaka lies 143 Kilometres from Kampala. In 2013, more than third of Uganda’s population lived below the poverty line of US $ 1.90 [16, 17]. Studies undertaken in Masaka indicate that caregivers of orphans in this area struggle with insecure employment and provision of food, a challenge to providing effective care to such vulnerable children [18]. Many of the caregivers use social networks to take care of the children though this also comes with challenges due to the changing family and kinship systems in the country. Many families and the kinship have felt the brunt of HIV/AIDS care and their reliance to support AIDS orphans has weakened [19, 20]. Due to the lessening family ties, some children sleep in different households seeking for care [20]. Some of the caregivers may not be biological relatives but only willing community members and this raises questions as to how these orphans meet their psychological and bonding needs within these “new and fluid” family systems [20]. The caregivers and orphan children still suffer stigma and many of the of the orphans struggle to secure emotional support within the caregiving families [20]. In addition, caregivers struggle with poor health yet health care access is limited in most of the remote areas in Masaka [18].

ART is one of the main child and adolescent care programs across Uganda and both government and private sectors agencies are heavily involved in its provision. Uganda pediatric care guidelines on ART recommend it for all HIV-infected children younger than 15 years regardless of CD4 cell count [1]. The agencies selected for inclusion in this project offered both ART, and mental health care for children and adolescents.

This qualitative sub-study was undertaken in Masaka district at semi-urban/rural sites (TASO and Kitovu Mobile). These two agencies provide ART and mental health care for children and adolescents. The district lies in central Uganda and is 119 KMs away from the capital city, Kampala. Masaka district has an estimated population of 300.000 and is largely rural with a few urban confinements. The main source of income in Masaka district is from agriculture. Majority of the population are from the G*anda* tribe. It is one of the districts in Uganda that were heavily affected by the HIV/AIDS pandemic for over two decades.

### Study participants

All data was collected by means of key informant interviews. A key informant has been described as an expert source of information [21] and because of their personal skills or position in the society/community they are able to provide deeper insight on a subject being investigated [16]. In this study we targeted health care professionals that were directly interacting with children and adolescents and others that were in service management of HIV/AIDS/mental health services in the study district.

#### Study tools

A key informant guide was developed and administered during the interviews (see Additional file 1). Our key informants were requested to focus on children that had been on ART for at least 3 years and have a mental distress (possibly having a long experience of seeking services for both HIV and mental distress). We aimed at 3 years of enrollment on ART because due to poor adherence (and other factors) some of these children on ART might have full-blown AIDS [1] and therefore visit the health facilities quite regularly (as compared to those CD4 [T cell test] levels, few symptoms and who were newly enrolled for ART). The children in the study communities are faced with a range of conditions such as hunger, clothing, violence from family members, crime (e.g. drug abuse), homelessness and lack of schooling opportunities. All these conditions are common at primary health care (PHC) level and are precipitators of mental distress [22]. Our study was conducted at PHC level. All our informants had been trained in WHO Mental Health Gap Action Program Intervention Guide MhGAP (http://www.who.int/mental_health/mhgap/mhGAP_intervention_guide_02/en/). As a national program, many of the informants can recognize mental distress, but are not formally trained in diagnosing and consequently refer patients, who are struggling severely, further on. Some of the key questions asked in the study included: How long have you been working in the field of HIV? Have you had any previous training in mental health? What are your current beliefs as a health care professional on children that have been on ART for a long time and have a mental distress? What are the barriers to treatment seeking for children with a double burden of HIV/AIDS and mental distress?

### Data collection procedure

All data was collected within a period of 1 month by the first author and a research assistant. All study informants were interviewed in their offices of work. Each interview lasted between 40 and 60 min. All interviews were conducted in English and transcribed by a research assistant. After transcription, the interviews were checked by the first author for quality and consistency.

### Data analysis

All data was collected and analyzed using content thematic analysis [23]. This approach is deemed suitable for analyzing multifaceted, important, and sensitive phenomenon [23]. HIV/AIDS and mental distress are regarded as sensitive issues in the study communities. Content thematic analysis involves breaking the transcribed interviews into small units, subjecting them to descriptive treatments [23, 24], and then organize them into themes and subthemes. For example, the theme abject poverty is broken into subthemes such as lack of sufficient food to support care and treatment, lack of means of transport to health facilities, lack of funds to purchase drugs. As noted, content thematic analysis is suitable for researchers to employ a relatively low level of interpretation as compared to other traditions such as grounded theory, hermeneutic phenomenology for which a high level of interpretation would be required [23]. Vaismoradi and colleagues [23] noted that content thematic analysis is suitable for and answering questions such as “what are the concerns people have about an event? What reasons do people have for using or not using a service or procedure? [23]. In this study, we address the perceptions of health professionals about their concerns as to why children and adolescents with HIV/AIDS and mental distress may not seek treatment from formal health service systems (Table 1).

**Table 1:** Socio-demographic characteristics of study respondents

Characteristics	Key informants Male (N = 15)
Gender	
Male	5
Female	10
Age of respondents	
25–34 years	5
35–44 years	8
45–54+	2
Religion of respondents	
Catholic	4
Protestant	11
Education Level of respondents	
Medical specialists (pediatrics)	1
Medical officer	2
Clinical officer	2
General nurses	3
Mid wives	2
Health care managers	2
Counselors	3

### Ethics

Science and ethical approval were sought and granted from the Research and Ethical Committee of the Uganda Virus Research Institute, the Science and Ethical Committee of the London School of Hygiene and Tropical Medicine and Uganda National Council for Science and Technology. In order to secure informed consent, informants were briefed about the aims and objectives of this study and were assured of confidentiality. We do not use their actual job titles in reporting the results in this study as one of the ways of maintaining confidentiality.

## Results

We interviewed a total of N = 15 key informants, including one medical officer (pediatrics), two medical officers (general), two clinical officers, three general nurses, two midwives, two health care managers and three counselors. Ten [10] key informants were females and 5 males. Majority [11] were Catholics and only 4 were Protestants. Majority [8] were aged between 35 and 44 years. At analysis level, these socio-demographic factors did not have an influence on results.

We present our results under four types of barriers: the family related barriers, community related barriers, individual level barriers and lastly, health systems barriers.

### Family related barriers to treatment seeking

Health professional reported quite a number of family level factors that act as barriers to treatment seeking for both HIV/AIDS and mental illness:

#### Caregivers’ level of education

Informants suggested that educated caregivers are likely to seek medical attention as the first choice as compared to those whose levels of education are low or those that might not have gone to school at all.

*Actually I think, should I say, it depends on the level of education of the attendants, because if the attendants are aware that mental distress can be managed like other distresses are managed, then they will end up seeking medical attention. But where the level of understanding is low, most of them think of witchcraft and in most cases they end up seeking herbal or traditional attention and when it fails, in most cases they neglect the individual (Clinician Kitovu Hospital).*

There are observations by our informant above that education is likely to affect the choice of seeking treatment where the first choice is a formal health system.

#### Lack of sufficient food to support care and treatment

Informants reported that impoverished families might not have enough food to support care and treatment. Yet, for people with HIV/AIDS, food is fundamental in improving their nutritional status.

*Now when you go to the home, you will be surprised with what is happening in their [home]. There is no food; no one is helping, and such things. (Official, Kitovu Mobile)*

*Yes, actually what we used to do in the past, only that funding has reduced a bit; we used to give them food. Because you would initiate someone on the drugs and they ask you, but now you have given me these drugs, where am I going to get the energy to take these drugs? I have spent over three days without a proper meal. (Clinician Kitovu Hospital)*

#### Lack of transport means to the health facilities

Children and adolescents with HIV/AIDS need a lot of medical attention for various ailments. Those with mental distress may also require regular medical review. This means that they need regular transport to the health facilities. Our findings indicate that quite a number of families may not afford transport charges for public transport to health facilities.

*“… distance is an issue. Because even if here I identify an adolescent with mental health issues that require an expert, we are lucky to be located within the referral hospital; but there are some issues that may be beyond and may require some to travel some distance, maybe going to Butabika [the national referral hospital for mental health], and every time you mention that, some will say. “I don’t have the money after all the child can still eat, play around”. So it’s a non*-*issue” (Medical officer, TASO Masaka).*

Health facilities in Uganda are quite distant with average distance of over 5 km in most rural areas. However, in many rural areas, the distance may be longer. The transport charges to the nearest health facility may not be affordable to the poor and this hinders their possibility of accessing formal health services.

#### Failure to buy drugs

It was noted during interviews that some families with children and adolescents with HIV/AIDS and mental distress could not afford the cost of drugs.

*Some children are ever sick and this drains family resources. Such families cannot afford the cost of drugs (Official, Kitovu mobile).*

Failure to purchase drugs was reported also common among child-headed households, which may not have any source of income.

*You find children looking after fellow children and how can such a family afford the cost of care for HIV/AIDS and mental distress? And, moreover mental health drugs are very expensive in this country (Nursing officer, TASO Masaka).*

It was also reported that it is common to find caregivers that also have HIV/AIDS and therefore do little or no productive work. There are also families whose resources have been drained because of constantly seeking of medical care for children/adolescents with HIV/AIDS and mental distress.

#### Family fatigue

Due to financial and psychological stress, which families with children and adolescents with HIV/AIDS and mental distress go through, some caregivers may give up and renounce their role to provide care.

*Yeah, biological or not, I think this is likely to happen also with those adolescents who were diagnosed and started taking ARVs at a much younger age. If you look at the one who has been taking medicine for 5* *years and you compare with someone who has been taking for 2 or 3* *years, the caregivers also burn out. Moreover, they will start saying: “After all, this child is big enough. He or she is pretending.” In addition, thereafter even coming to hospital will not be a priority anymore. And other issues will come in like: “After all, you don’t even do well in school”. Thus, those issues come in (Medical Officer, TASO Masaka)*

Others may also be struggling with HIV/AIDS or be old and therefore may not feel strong enough to look after their grandchildren.

*When the mother dumps them [sick children], the grand parents also get frustrated over time and they say, “This is not my child”! I am also taking care of mine [her/his biological children that are still under his/her care]! This could be a family where the grandmother and the grand father are all HIV positive and they are on Antiretroviral Therapy. So they tend to ignore their say, 5*-*year old grandchild who cannot make any decisions (Medical Officer, TASO).*

It seems quite clear from the views above that the double burden of HIV/AIDS and mental distress in an environment with limited resources stretches the notion of social care within the extended family to the brink of collapse and this consequently becomes a barrier to treatment seeking.

#### Community related barriers

In the section above, we have presented the family related barriers to treatment seeking. In this section, we deal with community related barriers that include community stigma, lack of support to families and children with HIV/AIDS and mental distress as well as violence to children with HIV/AIDS and mental distress.

#### Stigma affecting adherence and willingness to seek treatment

Informants reported that community stigma that is associated with having HIV/AIDS and mental distress is quite high and likely to be a barrier to adherence for both HIV/AIDS and psychiatric drugs:

*Stigma could also be there. Because now you see, we separated these clinics in such a way that once a child becomes 10* *years and above, we transfer them to the adolescents’ clinic. When they are still young, there is no stigma but when they become an adolescent that is when they start getting stigma… they start to understand that there is a problem. (Nurse, TASO, Masaka)*

The nurse seems to refer to self-stigma in this quote, as she mentions the developing understanding of the child. The children have to deal with a double burden of stigma for both HIV/AIDS and mental distress.

*Both of them attract stigma and children have to juggle both, the one for HIV and the one for mental distress here at the health unit and in community places such as schools (Medical, Officer TASO)*

#### Community failure to support families

Quite a number of informants noted that some families where struggling without community support even in child-headed families, yet community support is normatively supposed to be accorded to such children.

*You find a twelve*-*year old, or thirteen*-*year old, alone at home, ask them where the parents are, the parents died, then the other siblings are in Kampala and these ones stayed at home in Kyazanga. The child that stays at home is not regularly assessed. Because the money is not there and the community does not help. (Medical Officer TASO, Masaka)*

The community behavior depicted above is quite contrary to the expected social role of the community as it is supposed to guarantee the welfare of such vulnerable children.

#### Community violence

Again, in contrast to the social roles expected from the community, in addition to failure to support these children, despite their HIV/AIDS and mental health status, they in addition can be mistreated by the community. For example, it was reported that sometimes community members could unleash violence on them in disregard to their rights as vulnerable children. A Nursing officer from Masaka reported that these children are tempted to wander around as they have nothing to survive on in their homes. In these cases the community is not ashamed of using violence over even small mistakes.

*They can still be tied [on ropes] even when they are very weak due to long term distress. They can be beaten over little things if they wander around in the community. The community fails to remember that these are special children.* (Nursing officer, TASO Masaka)

#### Individual level barriers

As seen from the sections above, numerous barriers from the family and community hinder treatment seeking. Consequently, these barriers impact negatively on the functional capacity of the children (e.g. psychologically and socially) and this affects their willingness to seek treatment.

#### Exhaustion/the ‘I am tired’ concept

It was reported that in some situations children describe themselves as tired. They tend to look at their future as precarious and get into resignation.

It was for example, noted that within this precarious situation of resignation, they may start to ask existential questions as: “Why is it me who has HIV/AIDS but no other children? Why is it me who takes a tablet daily?” And they may blame their parents for their plight:

*…yes, for her she is positive! And she sees the elder brother is negative and those who follow her are negative. So she told me this…I told my mother, I am so angry with you! Why should I be positive? You did not give it to the other twin, you did not give it to the elder brother, and even the followers”! So, I saw that girl is [psychologically] disturbed. But she is coping (Nursing Officer TASO, Masaka)*

Despite her existential struggles and blame to her mother, this girl seems to cope even though she is psychologically troubled. It remains to see whether she will be able to maintain this ability to cope in the end.

Another medical officer describes a process, which goes from realizing their condition to blame the parents and the perception of being different from peers:

*Well, it always starts with a blame game. They always start with blaming the parents, school schedule; they complain that they did not know why they are taking the drugs. Now that they know, they start to blame the parents. They also talk about the inability to fit among their peers. Their peers always notice the hospital visits and that is the most disturbing thing (Medical Officer TASO, Masaka).*

Due to lack of psychological support, the children start also to doubt whether they are still useful to themselves, family and others *“they develop this feeling that they might not have value to self, others and family” (Offical, Kitovu Mobile).* The children seem to have lost the trust in themselves and their self-esteem gets demolished.

### Lack of knowledge

Especially those children and adolescents that are not part of family systems may not realize that they have mental distress and may not have information on where to go for treatment. It was reported that street children and orphaned children without caregivers are the common victims here.

*And sometimes, I am looking at street children as a common example here. Because once they start misbehaving then they can get HIV. However, sometimes they could not be having the knowledge of where to go and that is a hindrance. In addition, sometimes they may think that they may not be accepted at the health facility (Nursing official, TASO Masaka).*

Lack of knowledge among street children here seems to be paired with an anxiety of being an outcast of society and not accepted for treatment. In child-headed households the participants more pointed at sheer lack of knowledge.

*Now like you are HIV and then you are adolescent, of course most of the children we are dealing with are already orphans. May be they are even family heads in their homes. So they will not even know if they have a problem that need to be addressed (Official Kitovu, Mobile).*

### Health systems barriers

Ideally, in the event that family and community systems are faced with insurmountable challenges; one would expect the formal services to become the shock “absorbers”. However, our findings indicate that the health system in the study area was also experiencing quite a number of challenges. These included limited capacity to recognize mental distress, overwhelming burden from other diseases and conditions, institutional stigma and general lack of interest in mental health among health workers, understaffing, lack of service integration, and lack of policy guidelines on HIV/AIDS and mental health as well as frequent drug stock outs.

#### Limited capacity to recognize mental distress

Our informants reported that some of the health workers could not recognize mental distress and therefore were not comfortable attending to children with mental distress.

*Even as a medical officer, my skills to assess mental distress, I wouldn’t say they are the best, because we cover that element in school, but I notice that when we go to do our internship, that it is not so much incorporated. We don’t rotate in psychiatry so much. So we don’t really do that and for the lower cadres, it becomes quite challenging and frustrating and it is a lot easier for the attending clinician to just ignore that. (Medical Officer, TASO Masaka)*

#### Overwhelming diseases burden from other diseases and conditions and lack of interest

The health system has to deal with quite a number of diseases that have high mortality and morbidity in the study areas within limited resources. These are likely to shift the priority way from mental distress.

*You simply can’t dig deep enough to get to the bottom of the issue. We also tend to focus more on life threatening conditions and tend to think that the rest will be handled by someone else. So it also comes back to us because so many are waiting [in the line for treatment], so you want to work on people very fast; you only assess life threatening conditions. (Medical officer TASO, Masaka)*

Some informants also stigmatized the mental health sector and reported to have low interest in managing mental distress, especially in a context with other life-threatening conditions

*Yes, the care burden is also too much. I understand that most clinicians have their field of interest. And sincerely speaking, many of us are not interested in managing mental distress and to me, that would create a burden in managing them. And for the very few that are interested, they are overwhelmed by the number of patients with other conditions. So you are taken up by that number. (Clinician, Kitovu hospital)*

Together with lack of knowledge and interest anxiety was pointed at as a factor, which would keep the professionals away from people with mental problems:

*And with mental distress you need to probe a lot to reach at a diagnosis, which is actually time consuming. And the risks involved in handling a mental client;because a mental client can easily box you. (Clinician, Kitovu hospital)*

#### Barriers to the establishment of child and adolescent friendly services

Many of these factors were related to the health system being not tailored to adolescent needs.

#### Stock outs discourage service use

There were also reports of shortage of drugs and this could turn away quite a number of clients.

*Because they usually say that there are no drugs. Once they go there once and they don’t get drugs, they take it as if it is always like that that there will be no drugs. But still, we encourage them to attend the health centers near to them. (Nursing Officer, TASO)*

#### Understaffing

Many of the health facilities where reported understaffed and this creates a challenge in providing child and adolescent friendly services.

*You know, staffing challenges usually bring in a gap. Because we have counselors who are stationed there [in the clinics]. When it comes to the medics, we are a little bit limited by our numbers. Like yesterday you found me there, I was bridging the gap [because another medical officer was not around and yet at the same time he is the administrator]. (Medical Office TASO, Masaka)*

### Narrow services available/and lack of integrated care

Due to lack of service integration, children and adolescents have to seek services at multiple service centers.

*….it is very difficult for me to get a patient and then I say that this patient seems to have a mental problem. These programs are running kind of parallel. You see, we do not have the knowledge of mental distress in HIV positive children or even adults. So we have been having parallel programs and the focus of the health workers [on mental health] has been low! I think we should be able to deal with this problem. (Official Kitovu mobile)*

#### Lack of policy guidance on integration and management of mental disorders with other physical conditions

Some informants reported lack of policy guidelines on integration and management of mental disorders with other physical conditions. Yet for other health conditions such as HIV/AIDS and Malaria, guidelines are available to help lower cadre staff. Without these guidelines, some health workers seem not to be confident to manage mental health conditions that are comorbid with other physical problems.

*… I think it is really very important to kind of try to have, like most of these policies change. But I think as we continue to have more evidence, it is time to begin to think of having a comprehensive package even in policy development. Because you will find for example the first HIV policy we made never even had TB, and now TB came. So I think it is really important that we begin to think around comprehensive kind of policy development, whereby we are able to have something that addresses a little bit of everything [including mental health]. (Official, Kitovu, Mobile2)*

## Discussion

This study is the first study in Uganda, Africa and low-income countries to unravel perspectives of health care professionals on the barriers to treatment seeking for children with HIV/AIDS and mental distress. Our focus was those children who have been living with HIV/AIDS for a long time and have developed a mental disorder. As reported by our informants and as noted by Nelkin et al. [25], the impact of the HIV/AIDS pandemic has had long-term consequences for the individual, family and society social institutions. Several studies [26, 27] have noted the harsh environment in which the HIV/AIDS orphans live and, this environment becomes even harsher for those children that might have HIV/AIDS and mental distress. As indicated by the health professionals in this study and in other studies [28], many of these families live without adequate food; lack income to meet medical requirements of the family and transport costs to the health facilities; caregivers have low education levels and have safety related needs. What has been lacking in these studies is information as to whether these needs constrain treatment seeking for children with both HIV/AIDS and mental distress. It is quite clear from our findings that the care burden becomes bigger for families with children with HIV/AIDS as compared with other families who may not have children with HIV/AIDS. There is evidence that this burden grows bigger when children with HIV/AIDS develop mental distress [28]. The families are likely to be impoverished the more [29]. Moreover, community based interventions addressing family economic empowerment will be needed in such a context.

We also see community stigma towards the children that have mental distress and lack of respect for their rights as a vulnerable group. Public health programs on community stigma on HIV/mental health should address issues of children rights.

The health systems reported a long list of challenges. Many of these barriers (e.g. inadequate staff, shortage of drugs, low motivation of staff etc.) have been reported in related studies on barriers to treatment seeking [29, 30]. The major contribution of this study here is that the findings pertain to HIV/AIDS and mental distress. There is increased interest in Uganda and the LMICs to integrate mental health into primary health. These barriers need to be addressed, if adolescents with HIV/AIDS and mental health are to be effectively integrated into primary health care. Some of the resources meant for integration of mental health into primary health care can be used to address these barriers.

Orphans with HIV and mental distress, due to long battles with HIV/AIDS, were reported to be in a state of self-resignation and general apathy [31]. Children development models that are well tailored to this specific and vulnerable group will be vital. These need to be part of community based programs on HIV/AIDS and mental health in the study communities; which at the moment are lacking or not well developed in the study communities.

## Limitations of the study

It would have been important to triangulate information in this this study by interviewing key informants (service providers), children and adolescents (as service users) caregivers (who regularly stay with children and adolescents) and community leaders as (gatekeeping role for these children at community level. By doing this, we would have unraveled different perspectives to barriers to seeking treatment for formal health services among children and adolescents with HIV/AIDS and mental distress in the study area. The current study only has viewpoints from service providers. However, given the long experience of service providers in handling such children, we managed to secure useful information on the study subject. Future studies should address this study limitation.

## Conclusions

Understanding barriers to treatment seeking will deepen on the understanding of the impact of the HIV/AIDS carnage on the individual, family and community systems. The negative impact of the disease to family and community systems has wiped away the informal social systems of care. Stigma associated with mental distress play a cross cutting role in weakening community systems to provide care. Though formal health services system play a major role in supporting the informal system in the study areas, their impact is hardly felt by the families in the study area.

## Supplementary information

**Additional file 1.** Chaka study.

## Data Availability

Selected sections of the data used in this study can be obtained from the first author on request. Full interviews will not be shared because of ethical reasons as required by the ethical boards in Uganda. The fully data sets are therefore not publicly available and if needed this can be discussed with Eugene Kinyanda.

## Abbreviations

HIV/AIDS: Acquired immune deficiency syndrome or acquired immunodeficiency syndrome
KIs: Key Informant Interviews
FGs: Focus Group Discussion
CD 4: T-Cell Count
