# Supplementary material for: Health care professionals’ perspectives on barriers to treatment seeking for formal health services among orphan children and adolescents with HIV/AIDS and mental distress in a rural district in central, Uganda
Source: Child Adolesc Psychiatry Ment Health. 2020 Jun 3;14:26. doi: 10.1186/s13034-020-00332-8 (PMC7271468; doi:10.1186/s13034-020-00332-8)
Supplement: Supplementary file 1 — Additional file 1. Chaka study. [file 13034_2020_332_MOESM1_ESM.docx]

**Figure S1**

**CHAKA STUDY**

**Key Informant Interview guide - Health workers for children with HIV/AIDS and Mental Distress**

•         Career history

•         Current responsibilities

•         What does your work currently involve?

•         Are there any changes in the kind of task you have to do?

•         How enjoyable is your work?

•         How do you feel about a CA-HIV?

•         What is your experience working with CA-HIV/AIDS?

•         How much of your work is related to CA-HIV also having mental distress?

•         In addition to HIV/AIDS, they may also have mental health/ behavioral problems. Have you experienced HIV infected children with mental health problems in your work?

•         What are the main concerns of such children and adolescents?

•        What are the main concerns of affected families?

•         What can be done to improve services for children and adolescents with HIV/AIDS who have mental health problems?

•         What do you think about the work you have been doing since you started working?

•         What do you think can influence people to seek/not seek professional help for mental health problems? Reasons for and against/where is help sought from/Challenges in help seeking for mental health problems

•         How can we improve services for the children/adolescents with the mental health problems?

•         Any other thing you find important but we have not asked about?
